# The impact of an innovative payment method on medical expenditure, efficiency, and quality for inpatients with different types of medical insurance: evidence from a pilot city, China

**DOI:** 10.1186/s12939-024-02196-2

**Published:** 2024-06-05

**Authors:** Kunhe Lin, Yunfei Li, Yifan Yao, Yingbei Xiong, Li Xiang

**Affiliations:** 1 Department of Health Management, School of Medicine and Health Management, Tongji Medical College, Huazhong University of Science and Technology, Wuhan, China; 2 Department of Neurobiology, Care Sciences and Society, Karolinska Institutet, Stockholm, Sweden; 3 HUST Base of National Institute of Healthcare Security, Wuhan, China

**Keywords:** Innovative payment method, Medical insurance, Medical expenditure, Medical efficiency, Medical quality, Health equity

## Abstract

**Background:**

Since 2020, China has implemented an innovative payment method called Diagnosis-Intervention Packet (DIP) in 71 cities nationwide. This study aims to assess the impact of DIP on medical expenditure, efficiency, and quality for inpatients covered by the Urban Employee Basic Medical Insurance (UEBMI) and Urban and Rural Residents Basic Medical Insurance (URRBMI). It seeks to explore whether there are differences in these effects among inpatients of the two insurance types, thereby further understanding its implications for health equity.

**Materials and methods:**

We conducted interrupted time series analyses on outcome variables reflecting medical expenditure, efficiency, and quality for both UEBMI and URRBMI inpatients, based on a dataset comprising 621,125 inpatient reimbursement records spanning from June 2019 to June 2023 in City A. This dataset included 110,656 records for UEBMI inpatients and 510,469 records for URRBMI inpatients.

**Results:**

After the reform, the average expenditure per hospital admission for UEBMI inpatients did not significantly differ but continued to follow an upward pattern. In contrast, for URRBMI inpatients, the trend shifted from increasing before the reform to decreasing after the reform, with a decline of 0.5%. The average length of stay for UEBMI showed no significant changes after the reform, whereas there was a noticeable downward trend in the average length of stay for URRBMI. The out-of-pocket expenditure (OOP) per hospital admission, 7-day all-cause readmission rate and 30-day all-cause readmission rate for both UEBMI and URRBMI inpatients showed a downward trend after the reform.

**Conclusion:**

The DIP reform implemented different upper limits on budgets based on the type of medical insurance, leading to varying post-treatment prices for UEBMI and URRBMI inpatients within the same DIP group. After the DIP reform, the average expenditure per hospital admission and the average length of stay remained unchanged for UEBMI inpatients, whereas URRBMI inpatients experienced a decrease. This trend has sparked concerns about hospitals potentially favoring UEBMI inpatients. Encouragingly, both UEBMI and URRBMI inpatients have seen positive outcomes in terms of alleviating patient financial burdens and enhancing the quality of care.

**Supplementary Information:**

The online version contains supplementary material available at 10.1186/s12939-024-02196-2.

## Introduction

Global healthcare systems face immense pressure, such as populations age and chronic diseases rise, driving up medical expenditure in both developed and developing countries [1]. Most policymakers now pursue strategies to maximize health value from scarce resources by enhancing quality, outcomes and affordability simultaneously [2–4]. However, effectively curbing expenditures while improving efficiency and quality remains a formidable challenge worldwide. China is no exception as it also strives to address these issues [5]. While China has made progress improving population health in recent years, a considerable quality gap with developed countries still exists [6]. Reforms aim to decrease health expenditures and increase efficiency without compromising quality of care [7]. Moreover, tackling these challenges sustainably amid limited means requires reducing inequality amongst patients through prudent reforms [8].

Amid fiscal constraints and rising health burdens, many perceive fee-for-service (FFS) as inflating expenditures by encouraging overtreatment. In response, countries now view innovative payment methods as mechanisms to better align provider incentives with efficient, high-value care delivery. Since the 1980s, countries worldwide have re-evaluated healthcare payment methods and expedited efforts to explore innovative models to realign provider incentives and control expenditures [9]. For example, Diagnosis Related Groups (DRGs) and bundled payments are widely adopted innovative payment schemes globally. In contrast to FFS, DRGs and Episode-based bundled payments consolidate all services within discrete episodes like hospitalization into lump sum reimbursement [10]. By paying based on case types rather than itemized expenditures, they transfer financial risk from payers to providers [11]. This makes providers accountable for both expenditures and outcomes, aiming to bolster value. Studies indicate that the introduction of DRGs has generally led to a decrease in average expenditure per hospital admission, but has also resulted in negative effects such as incentivizing an increase in the number of inpatients receiving treatment, even creating patients, inadequate patient care services, and code upcoding [12–15]. Episode-based bundled payments have demonstrated significant savings in average expenditure per hospital admission, but their impact on quality remains inconclusive [16–18]. Moreover, the number of inpatients receiving treatment under episode-based bundled payments may unintentionally increase, enough to offset or negate expenditure reductions or savings to medical insurance [19]. Without strict budgetary mechanisms in place, both of these schemes may lead to excessive expenditure growth [20].

The rise of innovative payment methods has accompanied valid concerns about potential negative impacts on health disparities if not designed carefully. There are concerns episode-based bundle payments could worsen inequities through unintended effects. For example, existing racial disparities in access to and services for profitable, high-volume lower extremity joint replacement surgery (LEJR) surgery - a key target of bundles - risk exacerbating under programs like comprehensive care for joint replacement model (CJR) in America [21]. The 2023 World Economic Forum report, named “The Moment of Truth for Healthcare Spending: How Payment Models can Transform Healthcare Systems”, warned any new payment methods could widening social health gaps without fairness goals [22]. Populational models like ACOs also prompted such cautions. For example, multiple studies highlighted ACOs could exacerbate challenges facing low-income and racial minority groups [23, 24]. This could occur through provider selection mechanisms that inadvertently encourage some to avoid riskier, costlier patients upon participation. Similarly, unintended patient selection pressuring only certain, less complex patients could negate intended impacts. Innovative payment methods must consider inequitable impacts and ensure vulnerable populations fairly benefit.

The Chinese government has demonstrated a strong commitment to exploring innovative payment methods as an alternative to FFS. Building upon experiences from various countries and regional pilot trials in China conducted from 2013 to 2019, the Chinese government initiated a nationwide selection of 71 Cities as pilot sites starting in 2020. These pilots implement an innovative payment method called Diagnosis-Intervention Packet (DIP), which combines global budget with case-based payment. In pilot cities, the global budget ceiling is determined based on different types of medical insurance. the global budget is allocated to the regional inpatient healthcare system (rather than individuals or organizations) to establish the annual budget cap. The DIP reform utilizes 3–4 years of citywide case data to extract data features for case grouping, forming a disease library known as DIP groups. Each DIP group’s expenditure (post-treatment prices) primarily consists of points and point value (PV). Points are obtained by comparing historical expenditure with the average of all cases or specific case averages. PV is generated based on the ratio of the current year’s budget to the total points generated in the region that year. The global budget adopted by China serves as a price control measure, with PV adjusted annually under budget constraints to ensure expenditures remain within the set limits for the year. The Chinese government aims to reduce medical expenditure, enhance efficiency, and improve quality through DIP.

However, as mentioned earlier, innovative payment methods pose a risk of exacerbating health disparities if health equity is not considered. China primarily implements social medical insurance, including Urban Employee Basic Medical Insurance (UEBMI) for those with jobs and Urban and Rural Residents Basic Medical Insurance (URRBMI) for those without jobs, farmers, and even impoverished individuals, with a national coverage rate exceeding 95% [25–27]. Disparities in benefits and their impact on population health equity between UEBMI and URRBMI have long been significant concerns for Chinese policymakers and researchers [28–30]. Some studies have shown differences in medical expenditure among different types of medical insurance and have found that these different insurance types result in varied hospital care for patients [31, 32]. This variability may contribute to health disparities among patients. However, since DIP is a reform oriented towards healthcare service providers, considerations regarding whether inpatients with different types of medical insurance are affected by health equity were not initially included in its formulation. Due to differences in contributions, benefit coverage, and medical expenditures between China’s UEBMI and URRBMI, the budgets set upper limits based on different types of medical insurance. In the implementation of DIP, there is a problem of adequate budgets for UEBMI and tight budgets for URRBMI. The budgets directly influence the PV of each DIP group, leading to different post-treatment prices for the same DIP group between UEBMI and URRBMI inpatients. Whether this exacerbates medical equity for inpatients with different types of medical insurance is a matter worthy of consideration.

Existing research on the DIP reforms’ impact on medical expenditure, efficiency, and quality has yielded mixed findings, with impacts varying locally. For example, one study found an 8.5% average expenditure per hospital admission increase but and a 3.6% decrease in postoperative complication rates, when comparing a reformed vs. non-reformed city [33]. Guangzhou City saw short-term success slowing spending growth [34], while Chengdu City witnessed decreased average expenditure per hospital admission increase and improved medical quality post-reform [35]. One research in Taian City suggested utilization decreased at primary hospitals with less efficient resource use [36]. The DIP’s role remains unclear due to inconsistent evidence. Additionally, studies often analyze entire cities rather than investigating differences between employee and resident insured populations. This is relevant to the health equity of different population groups.

City A was designated as one of the 71 national pilot cities for the DIP reform. We conducted interrupted time series analysis (ITSA) on variables related to medical expenditure, efficiency, and quality for inpatients with different types of medical insurance in City A. The study aims to analyze whether DIP impacts varied based on medical insurance type. We seek to address three key questions. First, did DIP effectively reduce expenditures, improve efficiencies and enhance quality overall? Second, did outcomes differ between inpatient groups with UEBMI vs. URRBMI? Third, is there a risk of exacerbating inequities between insurance cohorts? Through this analysis in City A, we aim to provide clearer insights into DIP’s effects on expenditure, efficiency, and quality for inpatients with different insurance coverage.

## Materials and methods

### Study setting

City A, situated in Jiangxi Province, Central China, boasted a GDP of 123.75 billion yuan in 2022, placing it ninth in the province. Its permanent population was 1.156 million. The city accommodated 165.7 thousand participants in UEBMI and 977.0 thousand participants in URRBMI. Social insurance coverage reached 98.8%. The city’s medical infrastructure comprises a three-tier medical service network encompassing 88 hospitals, staffed by 2600 physicians, 3125 nurses, and providing a total of 8063 beds.

In late 2020, City A was designated one of the 71 national pilot cities for the DIP reform. Following a year of preparation, the DIP reform was rolled out across all hospitals in 2022, encompassing a total of 1646 DIP groups. Each DIP group’s average expenditure per hospital admission is compared to the mean expenditure of all cases, and this comparison determines the points assigned to each DIP group. While the points for each DIP group remain fixed, the actual monetary payment, or post-treatment price, to hospitals is determined during the year-end settlement based on PV, as indicated in Eq. (1). Hence, hospitals are only aware of the points for each DIP group until the year-end settlement, without knowledge of the final post-treatment prices. It should be noted that the Pre-determined Regional Budget arrangements for the two insurance types differ due to diverging funding schemes. Eq. (1) is typically modeled separately for UEBMI versus URRBMI in most reform cities. By the end of 2022, the PV for UEBMI stood at 9.29, while for URRBMI, it was 6.98. Consequently, for the same DIP group, the post-payment price for UEBMI inpatients was 1.33 times higher than that for URRBMI inpatients.

1 $$\begin{array}{c}PV=\frac{Pre\_determined\, Regional\, Budget}{Point\ sum\ of\ all\ inpatient\ cases\ within\ a\ region}\end{array}$$

As shown in Eq. (2), the actual payment to hospitals from medical insurance not only depends on the total points for inpatient services provided by the hospital itself but also on the total points from other hospitals in the market. Furthermore, it is also influenced by various adjustment factors, including hospital ranking, case mix index (CMI), etc., to ensure the rationality of payment standards. Regulatory measures employ big data technologies to verify the correctness of ICD coding, the comprehensiveness of medical services, and the reasonableness of discharge case payments. Emphasis is placed on monitoring and preventing violations such as excessive coding, inadequate services, and recurrent hospitalizations. Consequently, impose penalties and determine the amount for violations. Based on this framework, a positive value indicates a surplus in payments compared to the payment standard, while a negative value indicates a deficit that hospitals need to compensate for themselves. Moreover, to prevent hospitals from shifting expenditures to patients, regulations stipulate that hospitals’ actual payments from medical insurance and inpatients must not exceed the final predetermined post-treatment prices.

2 $$\begin{aligned}Reimbursements & =PV \times Hospital\ Point\ Volume \\ & \times actual\ reimbursement\ rate\\ & \times adjustment\ factors - Penalty\ amount\\ \end{aligned}$$

### Study design and data sources

This study conducted an empirical analysis using claims reimbursement data from the Medical Security Bureau in City A, Jiangxi province, China. The data covered a total of 663,434 inpatient reimbursement records from May 2019 to June 2023 across the entire city. From the records, we obtained inpatients’ demographic characteristics (age, gender), admission and discharge times, type of social medical insurance, inpatient expenditure, out-of-pocket expenditure (OOP) due to inpatient care, and length of stay. Inpatients were matched based on their unique personal identification codes. The interval time between the current and previous hospitalization was calculated based on the same patient’s admission and discharge times to determine if the current hospitalization was a readmission within 7 days or 30 days of the previous visit. May 2019 was excluded from the actual analysis to ensure accurate assessment of readmissions within these timeframes. Furthermore, to focus specifically on the effects of DIP implementation, cases that fell outside of DIP were excluded from the analysis, such as COVID-19 cases, mental illness cases, and rehabilitation cases. Therefore, the actual sample size analyzed consisted of 621,125 inpatient reimbursement records from June 2019 to June 2023, including 110,656 UEBMI inpatient records and 510,469 URRBMI inpatient records.

We conducted separate analyses of changes and trends in medical expenditure, efficiency, and quality for UEBMI inpatients versus URRBMI inpatients following the DIP reform. Specifically, we evaluated pre- and post-reform trends to determine if the reform differentially impacted medical expenditure, efficiency, and quality of care delivered to these distinct inpatient groups with UEBMI versus URRBMI over time. Through this approach, our aim was to provide insight into whether the DIP reform may have influenced health equity between UEBMI and URRBMI inpatients.

### Outcome variables

The aim of this study was to investigate the impact and disparities resulting from the DIP reform on medical expenditure, efficiency and quality for two types of social medical insurance inpatients. This exploration provides insights into the effects of the DIP reform on health equity among these inpatient groups. Therefore, the outcome variables in this study encompass three dimensions. Medical expenditure variables, including average expenditure per hospital admission and OOP per hospital admission, illuminate the economic burden of hospitalization for different social medical insurance inpatients post-DIP reform. Medical efficiency variables, measured by the average length of stay, reflect the efficiency of hospital care provided to different inpatients covered by social medical insurance following the DIP reform, in terms of duration. Furthermore, medical quality measures, such as 7-day and 30-day all-cause readmission rates, shed light on the post-hospitalization medical quality for different social medical insurance inpatients post-DIP reform.

### Statistical analysis

Using SPSS 24.0 software, the outcome variables were reported as mean ± standard deviation (SD) and tested by t-test. The medical expenditure variables were skewed, which were subjected to a natural log transformation to normalize their distribution.

We used ITSA, a robust quasi-experimental method, to assess the impact of the DIP reform on medical expenditure, efficiency, and quality for two types of social medical insurance inpatients. Using Stata 14 software, we included the outcome variables in ITSA from June 2019–June 2023. DIP reform measures were initiated in January 2022. Thus, we designated this period as the reform boundary and utilized it to create an indicator variable. Specifically, we assigned the value of 0 to denote the pre-reform period from June 2019 to December 2021, and the value of 1 to signify the post-reform period spanning January 2022 to June 2023. We analyzed the outcome variables for different types of social medical insurance inpatients to assess the magnitude and direction of changes before and after the reform. The ITSA model was exploited as follows:

3 $$\begin{array}{c}{Y}_{t}={\beta }_{0}+{\beta }_{1}\times T+{\beta }_{2}\times {X}_{t}+{\beta }_{3}\times T\times {X}_{t}+ {\epsilon }_{t}\end{array}$$

In this context, ${Y}_{t}$ represents the value of the outcome variables at time point t. ${\beta }_{0}$ denotes the baseline level of the outcome indicator at t = 0. ${\beta }_{1}$ estimates the trend of the dependent variable over time prior to the DIP reform. ${\beta }_{2}$ indicates the immediate change in the outcome variables at the moment of the DIP reform. ${\beta }_{3}$ captures the change in slope following the DIP reform. Hence, ${\beta }_{2}+{\beta }_{3}$ reflects the slope post-intervention. The variable T corresponds to the time series values t, ranging from 1 to 49 over the study period, measured in monthly intervals. Before the DIP reform, ${X}_{t}$ takes the value of 0. ${X}_{t}$ takes the value of 1 after the reform. $T\times {X}_{t}$ represents an interaction term, which is 0 before the reform and T after the reform. ${\epsilon }_{t}$ denotes the error term, accounting for random errors.

Regression model fitting is performed using ordinary least squares segmentation, where the reform initiation acts as the breakpoint. This facilitates the examination of significant differences in the trends of regression coefficients before and after the reform. Considering factors such as seasonality and autocorrelation, we conducted the regression with Newey–West standard errors for autocorrelation and carried out a seasonal adjustment.

### Delay effect checks

Theoretical reasons for policy effect delays include time required for awareness, implementation, behavioral/cultural changes. Therefore, many real-world policy interventions do not produce immediate effects and instead their impacts may be delayed due to lag periods in transmission mechanisms. To robustly estimate such delayed effects, traditional time series analyses adopting a single true intervention point may be underpowered. We conducted ITSA with the outcome variables, using March 2022 and June 2022 as counterfactual intervention dates, in addition to the actual January 2022 policy launch date. We compared effect sizes between the true and false interventions. Larger impacts for the actual date would suggest true lagged effects.

## Results

### Descriptive statistics

Table 1 shows changes in medical expenditure, efficiency, and quality for UEBMI and URRBMI inpatients before and after the DIP reform. The average inpatient expenditure per hospital admission for UEBMI inpatients decreased from 9.00 to 8.88. For URRBMI inpatients, it decreased from 8.73 to 8.63. The OOP per hospital admission decreased from 7.73 to 7.66 for UEBMI inpatients, and increased from 7.61 to 7.62 for URRBMI inpatients. Before the reform, the average length of stay was 10.91 days for UEBMI inpatients and 9.00 days for URRBMI inpatients. After the reform, it was 8.58 days for UEBMI inpatients and 7.22 days for URRBMI inpatients. The 7-day all-cause readmission rate decreased from 8.77 to 6.55% for UEBMI inpatients, and from 8.65 to 6.52% for URRBMI inpatients. The 30-day all-cause readmission rate decreased from 21.95 to 21.05% for UEBMI inpatients, and from 20.02 to 19.49% for URRBMI inpatients.

According to the results of the t-test, several variables for UEBMI and URRBMI inpatients were significantly different before and after the DIP reform. The details are shown in Table 1. These variables for UEBMI inpatients included the average expenditure per hospital admission (t = 5.149, *P* < 0.001), the OOP per hospital admission (t = 2.089, *P* = 0.042), the average length of stay (t = 5.801, *P* < 0.001), and 7-day all-cause readmission rate (t = 3.504, *P* = 0.001). These variables for URRBMI inpatients included the average expenditure per hospital admission (t = 5.197, *P* < 0.001), the average length of stay (t = 6.872, *P* < 0.001), and 7-day all-cause readmission rate (t = 4.183, *P* < 0.001).

**Table 1:** Basic description

Variables	UEBMI				URRBMI		
	Before the reform (2019.6–2021.12)	After the reform (2022.1–2023.6)	t		Before the reform (2019.6–2021.12)	After the reform (2022.1–2023.6)	t
Ln (Average expenditure per hospital admission + 1)	9.00 (0.07)	8.88 (0.09)	5.149 ***		8.73 (0.06)	8.63 (0.06)	5.197 ***
Ln (OOP per hospital admission + 1)	7.73 (0.13)	7.66 (0.11)	2.089 **		7.61 (0.08)	7.62 (0.12)	-0.366
Average length of stay (day)	10.91 (1.63)	8.58 (0.60)	5.801 ***		9.00 (1.34)	7.22 (0.41)	6.872 ***
7-day all-cause readmission rate (%)	8.77 (2.32)	6.55 (1.77)	3.504 ***		8.65 (1.88)	6.52 (1.41)	4.183 ***
30-day all-cause readmission rate (%)	21.95 (2.91)	21.05 (2.29)	1.118		20.02 (2.34)	19.49 (2.29)	0.767

*Note* Data are presented as mean (SD)

The significance levels of 1%, 5%, and 10% are denoted by ***, **, and *, respectively.

### The ITSA results of outcome variables for UEBMI inpatients

**Fig. 1:**
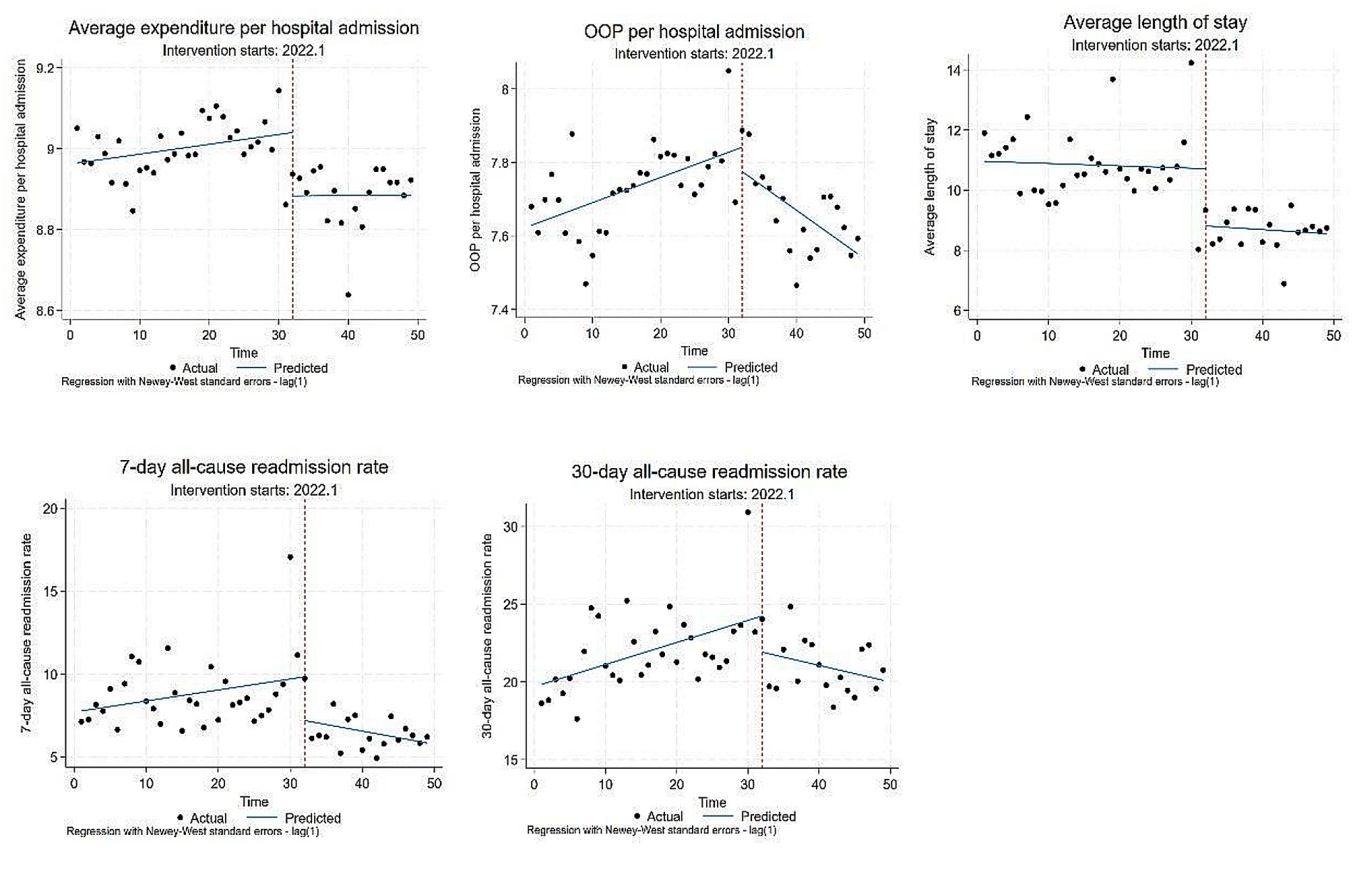
Changes in outcome variables for UEBMI patients after the DIP reform using ITSA

Figure 1 and Table 2 display the ITSA results of outcome variables for UEBMI inpatients before and after the DIP reform. The average expenditure per hospital admission for UEBMI inpatients showed an upward trend with a monthly slope of 0.2% (β_1_ = 0.002, *P* = 0.078) before the reform and immediately decreased by 15.7% (β_2_ = -0.157, *P* = 0.001) in the reform month. There was no significant change after the reform. The OOP per hospital admission for UEBMI inpatients showed an upward trend with a monthly slope of 0.7% (β_1_ = 0.007, *P* = 0.001) before the reform and exhibited a downward trend with a monthly decrease of 2.0% (β_3_ = -0.020, *P* < 0.001) after the reform. The average length of stay for UEBMI inpatients immediately decreased by -1.903 days (β_2_ = -1.903, *P* = 0.003) in the reform month. There was no significant change before or after the reform. The 7-day all-cause readmission rate for UEBMI inpatients immediately decreased by 2.641% (β_2_ = -2.641, *P* = 0.041) in the reform month, and exhibited a downward trend with a decrease of 0.146%/month (β_3_ = -0.146, *P* = 0.062) after the reform. The 30-day all-cause readmission rate for UEBMI inpatients showed an upward trend with a slope of 0.142%/month (β_1_ = 0.142, *P* = 0.011) before the reform. It immediately decreased by 2.327% (β_2_ = -2.327, *P* = 0.097) in the reform month and exhibited a downward trend with a decrease of 0.249%/month (β_3_ = -0.249, *P* = 0.010) after the reform.

**Table 2:** The ITSA results of outcome variables for UEBMI inpatients

Variables	Before the reform (2019.6–2021.12)				Reform instantaneous (2022.1)				After the reform (2022.1–2023.6)		
	β1	SE	t		β2	SE	t		β3	SE	t
Ln (Average expenditure per hospital admission + 1)	0.002	0.001	1.80*		-0.157	0.044	-0.002***		-0.002	0.003	-0.88
Ln (OOP per hospital admission + 1)	0.007	0.002	3.53***		-0.066	0.060	-1.10		-0.020	0.004	-4.54***
Average length of stay (day)	-0.008	0.026	-0.31		-1.903	0.595	-3.20***		-0.007	0.029	-0.26
7-day all-cause readmission rate (%)	0.066	0.055	1.20		-2.641	1.255	-2.10**		-0.146	0.076	-1.91*
30-day all-cause readmission rate (%)	0.142	0.053	2.67**		-2.327	1.373	-1.70*		-0.249	0.093	-2.67**

*Note* The significance levels of 1%, 5%, and 10% are denoted by ***, **, and *, respectively

### The ITSA results of outcome variables for URRBMI inpatients

**Fig. 2:**
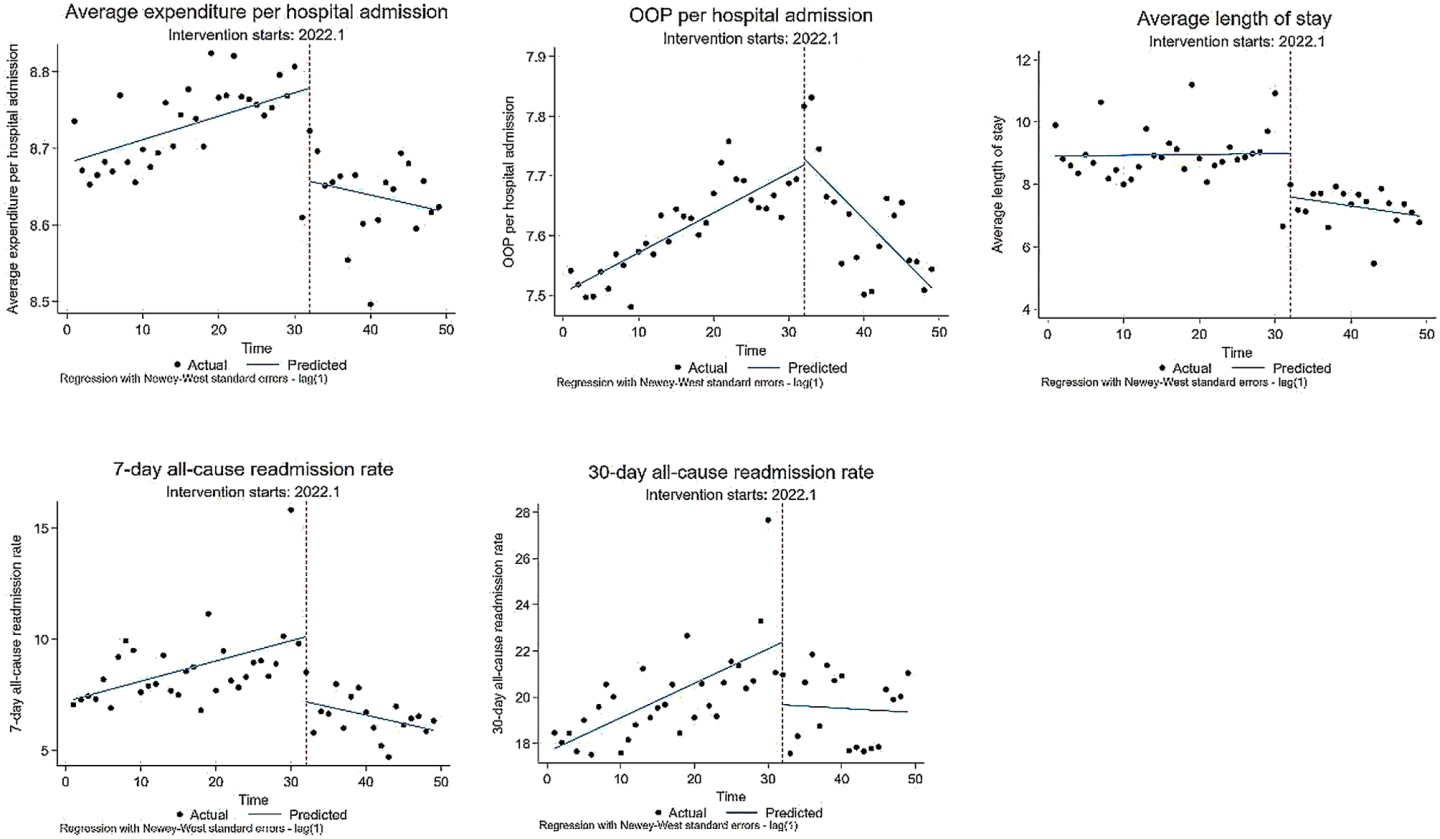
Changes in outcome variables for URRBMI inpatients after the DIP reform using ITSA

Figure 2 and Table 3 display the ITSA results of outcome variables for URRBMI inpatients before and after the DIP reform. The average expenditure per hospital admission for URRBMI inpatients showed an upward trend with a monthly slope of 0.3% (β_1_ = 0.003, *P* = 0.011) before the reform. It immediately decreased by 12.2% (β_2_ = -0.122, *P* = 0.003) in the reform month and exhibited a downward trend with a monthly decrease of 0.5% (β_3_ = -0.005, *P* = 0.010) after the reform. The OOP per hospital admission for URRBMI inpatients showed an upward trend with a monthly slope of 0.7% (β_1_ = 0.007, *P* < 0.001) before the reform and exhibited a downward trend with a monthly decrease of 1.9% (β_3_ = -0.019, *P* < 0.001) after the reform. The average length of stay immediately decreased by -1.394 day (β_2_ = -1.394, *P* = 0.003) in the reform month and exhibited a downward trend with a decrease of 0.039 day/month (β_3_ = -0.039, *P* = 0.086) after the reform. The 7-day all-cause readmission rate for URRBMI inpatients showed an upward trend with a slope of 0.091%/month (β_1_ = 0.091, *P* = 0.025) before the reform. It immediately decreased by 2.929% (β_2_ = -2.929, *P* = 0.002) in the reform month and exhibited a downward trend with a decrease of 0.166%/month (β_3_ = -0.166, *P* = 0.002) after the reform. The 30-day all-cause readmission rate for URRBMI inpatients showed an upward trend with a slope of 0.149%/month (β_1_ = 0.149, *P* < 0.001) before the reform. It immediately decreased by 2.718% (β_2_ = -2.718, *P* = 0.018) in the reform month and exhibited a downward trend with a decrease of 0.167%/month (β_3_ = -0.167, *P* = 0.040) after the reform.

**Table 3:** The ITSA results of outcome variables for URRBMI inpatients

Variables	Before the reform (2019.6–2021.12)				Reform instantaneous (2022.1)				After the reform (2022.1–2023.6)		
	β1	SE	t		β2	SE	t		β3	SE	t
Ln (Average expenditure per hospital admission + 1)	0.003	0.001	2.65**		-0.122	0.038	-3.18***		-0.005	0.002	-2.68**
Ln (OOP per hospital admission + 1)	0.007	0.001	7.59***		0.011	0.050	0.22		-0.019	0.004	-5.17***
Average length of stay (day)	0.003	0.019	0.14		-1.394	0.436	-3.20***		-0.039	0.022	-1.75*
7-day all-cause readmission rate (%)	0.091	0.039	2.32**		-2.929	0.913	-3.21***		-0.166	0.050	-3.36***
30-day all-cause readmission rate (%)	0.149	0.038	3.89***		-2.718	1.107	-2.45**		-0.167	0.079	-2.12**

*Note* The significance levels of 1%, 5%, and 10% are denoted by ***, **, and *, respectively

### Delay effect checks

Table S1 and Table S2 in Additional file 1 present the analysis results across two false intervention dates, including March 2022 and June 2022, to test delayed effects. The results showed that the DIP reform was not significantly associated with immediate changes in the month of reform implementation or changes in trends afterwards for most outcome variables. For the few variables where changes were observed, the magnitude was smaller than the impact of DIP implementation in January 2022. By analyzing different assumed intervention points, the results showed that the reform might have exhibited some degree of delayed effects. However, it also demonstrated that the changes following the January implementation were more prominent and the impact was greatest. Therefore, it can be concluded that January was the true time point of impact from the DIP reform. This helps validate the research design and makes the conclusions more robust.

## Discussion

We analyzed the impact of the DIP reform on medical expenditure, efficiency, and quality for UEBMI inpatients and URRBMI inpatients, using City A, from Jiangxi province, as a case study. The results indicated that the average expenditure per hospital admission for UEBMI inpatients showed an upward trend with a monthly slope of 0.2% before the reform. After the reform, the average expenditure per hospital admission did not significantly differ but maintained the upward pattern. Conversely, the average expenditure per hospital admission for URRBMI inpatients switched from an increasing pre-reform trend to a decreasing post-reform trend, declining by 0.5%. The average length of stay for UEBMI showed a sharp decrease only in the month of reform, with no significant changes before and after the reform. In contrast, there was a noticeable downward trend in the average length of hospital stay for URRBMI after the reform. The OOP per hospital admission, 7-day all-cause readmission rate and 30-day all-cause readmission rate for both UEBMI inpatients and URRBMI inpatients showed a downward trend after the reform.

The average expenditure per hospital admission for URRBMI inpatients shifted from an upward pre-reform trend to a downward trajectory post-reform. In contrast, expenditures for UEBMI inpatients continued the ascending pattern observed prior to the implementation of DIP. Following the DIP reform, the profitability of hospital receipts from medical insurance payments hinges on the variance between actual medical expenditures and the subsequent post-treatment prices during the year-end settlement. As UEBMI fund in China is funded by employees who pay higher medical insurance premiums compared to those paid by unemployed individuals, farmers, and others contributing to the URRBMI fund [37, 38]. UEBMI is endowed with a more generous budget, resulting in higher post-treatment prices for UEBMI inpatients with similar conditions compared to URRBMI inpatients. When attending to URRBMI inpatients, physicians face budget constraints and a larger inpatient volume, leading to lower post-treatment prices. Consequently, they are compelled to control medical expenditures to ensure that actual expenditures remain below the post-treatment prices. Any excess must be absorbed by the hospital. In contrast, when treating UEBMI patients, physicians are more inclined to prioritize immediate benefits due to the higher post-treatment prices.

The changes in average length of stay under UEBMI and URRBMI following the DIP reform provide evidence supporting this perspective. Post-reform, there was no significant change in UEBMI inpatients’ average stay duration, whereas URRBMI inpatients demonstrated a decreasing trend. This suggests that URRBMI inpatients effectively reduce medical costs by shortening hospital stays to enhance medical efficiency, thereby facilitating a reduction in per admission medical expenditures. In contrast, UEBMI inpatients lack incentives for improving medical efficiency due to generous post-treatment prices. Moreover, by increasing medical expenditures, physicians can augment the point allocation during the subsequent year’s DIP group adjustment, thereby elevating the post-treatment prices for that DIP groups. Meanwhile, there is a declining trend in the average length of stay and average expenditure per hospital admission among URRBMI inpatients. We are concerned about the possibility of decomposing hospitalization, but this study has yet to verify it.

Prior to the DIP reform, a predisposition existed among Chinese physicians to preferentially admit UEBMI inpatients. The incongruent trends in the average expenditure per hospital admission between UEBMI and URRBMI inpatients are concerning, as they may exacerbate inpatient selection behavior among physicians. According to the theory of physician agency in health economics literature, financial incentives play a crucial role in shaping providers’ decision-making processes [39]. This preference may exacerbate the uneven distribution of medical resources, leading to the overutilization of medical services by UEBMI inpatients while URRBMI inpatients may face insufficient access to medical care. This undermines the equitable access to medical care for residents without employed, particularly those in rural and impoverished communities within the city [40]. Innovative payment method represents a crucial pathway to effectively control hospital expenditures through the rigorous enforcement of budgetary constraints. Clearly, the global budget payment should impose stricter parameters on budget amounts [41]. Taking the Netherlands as an example of strict budgetary management, where global budget payment regulations ensure more effective financial management and control over medical expenditures [42]. At the same time, it is necessary to balance the annual budget caps for UEBMI and URRBMI, gradually eliminate the differences in post-treatment prices between UEBMI and URRBMI, and promote fairness in treatment for both UEBMI and URRBMI inpatients.

Encouragingly, our findings showed that following implementation of the DIP reforms, OOP per hospital admission transitioned from a previously rising trajectory to a downward trend. This post-reform shift in OOP per admission represents a positive development that may help alleviate financial burdens for UEBMI and URRBMI inpatients. The decline in the OOP per hospital admission for both UEBMI and URRBMI inpatients primarily stems from the DIP reform. Under this reform, post-treatment prices now consider medical expenditure in addition to insurance reimbursement prices. This end-of-year price adjustment ensures that the post-treatment prices encompass both the inpatient’s OOP and the medical insurance payments to the hospital. Consequently, the combined sum of the inpatient’s OOP and the hospital’s medical insurance reimbursement for this admission must not exceed the post-treatment prices determined by the DIP group. There is an inverse relationship between the OOP of inpatients and the amount paid by medical insurance to the hospital for this admission. When physicians primarily increase inpatient expenditures by raising OOP payments from inpatients, it effectively reduces the reimbursement amount provided by medical insurances to the hospital. Not only does this strategy fail to enhance profits by offloading expenditures onto inpatients’ OOP, but it also exacerbates the financial burden on those inpatients. This unequitable approach does not truly achieve the goal of boosting revenues. Physicians must consider the economic strain this imposes on inpatients and the increased sensitivity inpatients have towards medical expenditures, which may result in dissatisfaction with medical care.

Furthermore, due to the implementation of performance-based payment following the introduction of DIP in City A, both the 7-day and 30-day all-cause readmission rates showed a downward trend. The enhancement of medical quality for both UEBMI and URRBMI inpatients is notable. This finding is consistent with results from other studies. Performance-based payment systems can effectively oversee service providers and guide them in improving service delivery behaviors according to performance objectives [43]. For example, with the introduction of The Hospital Readmissions Reduction Program (HRRP) in the United States, a performance-based payment model, both safety-net and non-safety-net hospitals exhibited a parallel downward trend [44]. In City A, hospitals that exceed the past readmission rate standards will have their quality assurance deposits deducted as penalties during the year-end payment process conducted by the Medical Security Bureau. This increases the expenditures of non-compliance, shifting the incentive from economically benefiting through maliciously expanding service volume to incurring economic losses. This also addresses our concern regarding doctors potentially restricting medical services for URRBMI inpatients to control expenditures, thereby risking a decline in quality. However, we remain concerned that inadequate quality supervision during DIP reforms in other cities may lead to physicians excessively controlling medical expenditures, resulting in reduced medical quality, particularly harming URRBMI inpatients and exacerbating health disparities. This suggests that regions implementing DIP reforms need to strengthen supervision and penalties on medical quality to prevent under-service behaviors driven by profit motives.

This study has strengths. Firstly, the data covered a total of 663,434 inpatient reimbursement records from May 2019 to June 2023 across the entire city. This ensures the sufficiency of data for evaluation. Secondly, all data came from the inpatient reimbursement records of the Medical Security Bureau in City A, which guarantees the quality of data. Thirdly, performing ITSA on outcome variables for both UEBMI and URRBMI inpatients enables a more effective investigation of their respective impacts before and after the DIP reform.

This study has several limitations. Firstly, it relies on data from a single source, obtained solely from one city. Secondly, it lacks control groups from non-reformed areas for comparison. Thirdly, due to its relatively short timeframe, it cannot fully capture the long-term impacts of the DIP reform. Lastly, the analysis only includes 5 variables, resulting in a lack of information regarding other potential variables.

## Conclusion

China has created an innovative payment method called DIP. In 2020, 71 cities across the country were selected for pilot implementation. DIP combines global budget payment, case-based payment, and performance-based payment, aiming to reduce medical expenditures, enhance efficiency, and improve quality. However, as the reform was still in an experimental stage, equitable inpatient care for those covered by UEBMI and URRBMI remained a concern to be adequately addressed. This study uses City A as a case study to separately analyze the medical expenditure, efficiency and quality for UEBMI and URRBMI inpatients. The findings show that the average expenditure per hospital admission and the average length of stay for UEBMI inpatients remained unchanged compared to URRBMI inpatients whose expenditures and days decreased, after the DIP reform. This trend raises concerns about potential prioritization of UEBMI inpatients by hospitals. Encouragingly, OOP per hospital admission and 7-day and 30-day all-cause readmission rates for both UEBMI and URRBMI inpatients showed a downward trend, indicating positive effects in relieving financial burden for inpatients and enhancing care quality.

### Electronic supplementary material

Below is the link to the electronic supplementary material.

Supplementary Material 1

## Data Availability

The data supporting the findings of this study are available from the Medical Security Bureau information system in City A. However, restrictions apply to the availability of these data, which were used under license for the current study and are therefore not publicly available. Data are however available from the corresponding author upon reasonable request and with permission of the Medical Security Bureau.
