# Supplementary material for: The impact of an innovative payment method on medical expenditure, efficiency, and quality for inpatients with different types of medical insurance: evidence from a pilot city, China
Source: Int J Equity Health. 2024 Jun 5;23:115. doi: 10.1186/s12939-024-02196-2 (PMC11151554; doi:10.1186/s12939-024-02196-2)
Supplement: Supplementary file 1 — Supplementary Material 1 [file 12939_2024_2196_MOESM1_ESM.docx]

**Table S1** Delay effect checks: intervention date (March 2022)

| **types of Medical insurance** | **Variables** | **Before the reform**  **(2019.6 - 2022.2)** | | |  | **Reform instantaneous**  **(2022.3)** | | |  | **After the reform**  **(2022.3 – 2023.6)** | | |
| --- | --- | --- | --- | --- | --- | --- | --- | --- | --- | --- | --- | --- |
|  |  | **β1** | ***SE*** | ***t*** |  | **β2** | ***SE*** | ***t*** |  | **β3** | ***SE*** | ***t*** |
| **UEBMI** | **Ln (Average expenditure per hospital admission + 1)** | 0.001 | 0.001 | 0.89 |  | -0.061 | 0.054 | -1.20 |  | 0.001 | 0.004 | 0.38 |
|  | **Ln (OOP per hospital admission + 1)** | 0.007 | 0.002 | 4.33*** |  | -0.074 | 0.050 | -1.34 |  | -0.014 | 0.004 | -3.45*** |
|  | **Average length of stay (day)** | -0.028 | 0.027 | -1.04 |  | -2.021 | 1.020 | -1.44** |  | 0.013 | 0.034 | 0.38 |
|  | **7-day all-cause readmission rate (%)** | 0.046 | 0.049 | 0.93 |  | -0.070 | 0.081 | -1.23 |  | -0.079 | 0.057 | -1.37 |
|  | **30-day all-cause readmission rate (%)** | 0.116 | 0.051 | 2.28** |  | -0.150 | 0.100 | -1.52 |  | -0.216 | 0.106 | -2.03* |
| **URRBMI** | **Ln (Average expenditure per hospital admission + 1)** | 0.002 | 0.001 | 2.08** |  | -0.044 | 0.034 | -1.25 |  | -0.002 | 0.002 | -0.80 |
|  | **Ln (OOP per hospital admission + 1)** | 0.008 | 0.001 | 7.03*** |  | -0.005 | 0.005 | -1.00 |  | -0.015 | 0.003 | -4.46*** |
|  | **Average length of stay (day)** | -0.012 | 0.020 | -0.60 |  | -1.148 | 0.451 | -2.54** |  | -0.022 | 0.027 | -0.82 |
|  | **7-day all-cause readmission rate (%)** | 0.059 | 0.042 | 1.42 |  | -2.574 | 0.988 | -2.60** |  | -0.131 | 0.050 | -2.62** |
|  | **30-day all-cause readmission rate (%)** | 0.115 | 0.427 | 2.70** |  | -0.118 | 0.130 | -0.87 |  | -0.150 | 0.095 | -1.57 |

Note: The significance levels of 1%, 5%, and 10% are denoted by ***, **, and *, respectively.

**Table S2** Delay effect checks: intervention date (June 2022)

| **types of Medical insurance** | **Variables** | **Before the reform**  **(2019.6 - 2022.5)** | | |  | **Reform instantaneous**  **(2022.6)** | | |  | **After the reform**  **(2022.6 – 2023.6)** | | |
| --- | --- | --- | --- | --- | --- | --- | --- | --- | --- | --- | --- | --- |
|  |  | **β1** | ***SE*** | ***t*** |  | **β2** | ***SE*** | ***t*** |  | **β3** | ***SE*** | ***t*** |
| **UEBMI** | **Ln (Average expenditure per hospital admission + 1)** | 0.001 | 0.001 | 0.14 |  | -0.054 | 0.054 | -1.10 |  | 0.011 | 0.002 | 0.31 |
|  | **Ln (OOP per hospital admission + 1)** | 0.006 | 0.002 | 3.43*** |  | -0.040 | 0.055 | -1.14 |  | -0.005 | 0.006 | -0.83 |
|  | **Average length of stay (day)** | -0.045 | 0.022 | -2.01** |  | -1.101 | 0.742 | -1.65 |  | 0.040 | 0.039 | 1.04 |
|  | **7-day all-cause readmission rate (%)** | 0.012 | 0.044 | 0.28 |  | -0.029 | 0.039 | -0.80 |  | -0.011 | 0.070 | -0.16 |
|  | **30-day all-cause readmission rate (%)** | 0.093 | 0.047 | 2.00** |  | -0.149 | 0.100 | -1.63 |  | -0.141 | 0.107 | -1.33 |
| **URRBMI** | **Ln (Average expenditure per hospital admission + 1)** | 0.001 | 0.001 | 0.78 |  | -0.046 | 0.037 | -0.90 |  | 0.004 | 0.004 | 1.17 |
|  | **Ln (OOP per hospital admission + 1)** | 0.003 | 0.001 | 6.40** |  | -0.034 | 0.037 | -1.11 |  | -0.008 | 0.004 | -2.13** |
|  | **Average length of stay (day)** | -0.026 | 0.017 | -1.54 |  | -0.865 | 0.486 | -1.78* |  | -0.008 | 0.036 | -0.21 |
|  | **7-day all-cause readmission rate (%)** | 0.028 | 0.038 | 0.74 |  | -2.322 | 1.073 | -2.16** |  | -0.081 | 0.063 | -1.28 |
|  | **30-day all-cause readmission rate (%)** | 0.094 | 0.037 | 2.55** |  | -2.114 | 1.318 | -1.53* |  | -0.081 | 0.118 | -0.69 |

Note: The significance levels of 1%, 5%, and 10% are denoted by ***, **, and *, respectively.
